# Cortex reorganization of *Xenopus laevis *eggs in strong static magnetic fields

**DOI:** 10.1186/1477-044X-3-2

**Published:** 2005-12-13

**Authors:** Daniel Mietchen, Jörg W Jakobi, Hans-Peter Richter

**Affiliations:** 1 Fraunhofer Institute for Biomedical Engineering (IBMT), St. Ingbert, Germany; 2 Department of Physics and Mechatronics, University of the Saarland, Saarbrücken, Germany; 3 Fachhochschule Gießen-Friedberg, Gießen, Germany; 4 Department of Physiology, University of the Saarland, Homburg, Germany

## Abstract

Observations of magnetic field effects on biological systems have often been contradictory. For amphibian eggs, a review of the available literature suggests that part of the discrepancies might be resolved by considering a previously neglected parameter for morphological alterations induced by magnetic fields – the jelly layers that normally surround the egg and are often removed in laboratory studies for easier cell handling. To experimentally test this hypothesis, we observed the morphology of fertilizable *Xenopus laevis *eggs with and without jelly coat that were subjected to static magnetic fields of up to 9.4 T for different periods of time. A complex reorganization of cortical pigmentation was found in dejellied eggs as a function of the magnetic field and the field exposure time. Initial pigment rearrangements could be observed at about 0.5 T, and less than 3 T are required for the effects to fully develop within two hours. No effect was observed when the jelly layers of the eggs were left intact. These results suggest that the action of magnetic fields might involve cortical pigments or associated cytoskeletal structures normally held in place by the jelly layers and that the presence of the jelly layer should indeed be included in further studies of magnetic field effects in this system.

## Background

The molecular processes governing the action of static magnetic fields on living systems remain poorly understood, partly because the experimental evidence is equivocal (reviewed in [1,2]). As for amphibian development, the hatching rate of embryos of the frog *Rana pipiens *subjected to the field of a 1 T permanent magnet was found to be reduced [3]. This stimulated further studies in the frog *Xenopus laevis *whose giant eggs with a diameter of about 1.3 mm have rendered it a popular model system [4,5]. The effects observed therein ranged from reduced tadpole pigmentation at 1 T [6] to cleavage plane alterations between 1.7 T and 17 T [7] to no anomaly at all [8-11].

A closer look at these results reveals, however, that magnetic field effects might at least be partially dependent upon a parameter that has so far been neglected (cf. Table 1): In general, effects were only observed if the mucous three-layered jelly coat (JC) surrounding the *Xenopus *eggs had been removed directly after fertilization (for which it is required [12]), while no effects resulted when it was left intact. The JC serves multiple functions, one of which is to glue the eggs to their substrate [4,5]. As this complicates cell handling, experimentors often remove the JC [6,10,13-16]. Since this represents an unphysiologlal interference with the cell state, the aim of our study was to shed some light on whether this custom might influence the action of static magnetic fields upon the egg.

**Table 1:** Correlation between magnetic field effects and the JC presence in eggs or embryos

Magnetic field effects and jelly coat				
Field^a^	Magnet^b^	Jelly removed	Effect	Ref.

0		no	no	[4]
0.25		no	no	[8]
1.0		yes and no^#^	more embryos abnormal	[6]
1.5		no	no	[9]
6.34		yes^c^	no	[10]
8		no	no	[11]
17		yes	cleavage plane reorientation	[7]

^a^Maximal field strength in T

^b^Types of magnets: E = electromagnet; SC = superconductor. Superscripts denote the application of field gradients, subscripts indicate radiofrequency pulsing as in magnetic resonance experiments.

^# ^The experiments were mainly performed on cysteine-dejellied eggs but jelly-coated embryos were mentioned to give essentially the same results.

^c^Eggs were dejellied not immediately after fertilization but about 1 h later. Description is ambiguous on whether field exposure started before or after dejellying.

### Experimental procedures

*Xenopus *females were maintained under physiological standard conditions [5], and all experiments have been carried out according to institutional ethical guidelines. Oocyte maturation continued and egg ovulation followed after injection of 500–1.200 IU (according to female size) of gonadotropin (HCG, Sigma-Aldrich, Germany) into the dorsal lymphatic sack [5]. After 8–12 h, fertilizable eggs were spawned directly into isotonic modified Earth's medium [17]. JC removal was achieved by lysis with 2 % of cysteine chloride (VWR International, Darmstadt Germany) in Barth's, at pH 8, for about 2 min [13] and the cells then rinsed intensively with Barth's.

For each experiment, freshly spawned fertilizable eggs from only one female were used, either dejellied or not. To expose eggs of the same batch to different field strengths at the same time, they were distributed in groups of roughly 150 to about 15 Petri dishes of 65 mm outer diameter that were placed in a 50 cm long rack with 24 equidistant storeys. The rack was then slowly (with about 3 mm/s) inserted into the shim system of the vertical superconducting magnet of a DMX 400 NMR spectrometer (Bruker, Rheinstetten, Germany). The magnetic field strength in the rack varied between 0.5 T and 9.4 T. Control eggs (zero field references) both with and without JC were placed approx. 8 m from the magnet (about 70 *μ*T Earth's field strength) under otherwise identical conditions. Fixation was carried out with 2.5 % glutaraldehyde in Barth's [5]. Temperature was kept at (21 ± 1)°C throughout the experiments.

### The Tennis Ball Effect (TBE)

**Figure 1:**
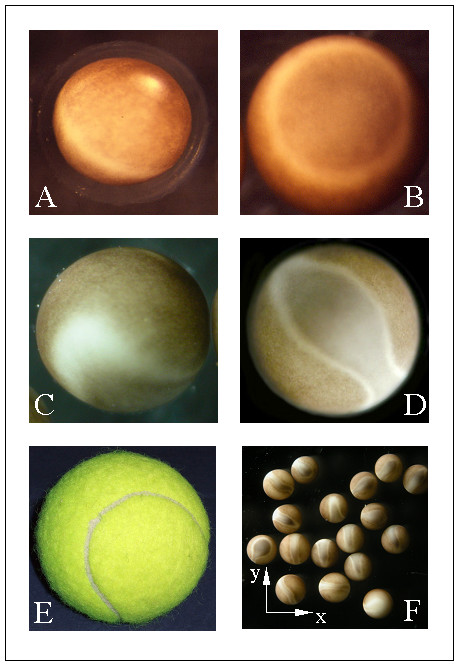
**Tennis Ball Effect in fertilizable eggs. **(A) Jelly-coated egg not exposed to the magnet. Note the position of the white equatorial line. The overall appearance and the pigmentation pattern are indistinguishable from the jelly-coated eggs exposed to the magnet and from the dejellied controls not exposed to the magnet (not shown). (B-D and F) Cysteine-dejellied eggs after exposure to the magnet, with altered cortical pigmentation. (B) Vegetal view of a late TBE I, with the equatorial line descended towards the vegetal pole. (C) Lateral view of an intermediate TBE II, showing the tongue that reaches out from the descended equatorial line. (D) Animal view of late TBE II, with the tongue from the equatorial line having reached the animal pole. (E) Tennis ball. (F) Orientation of the TBE in the magnetic field. The magnetic field's central axis (z) was perpendicular to the image plane. The images in (A), (C-D) and (F) were taken prior to and the one in (B) after fixation.

A typical control egg with intact JC and no exposure to the magnetic field is depicted in Fig. 1A, appearing as described in the literature [4]. Removal of the JC does not alter the egg's phenomenology, which is essential for the popularity of dejellying procedures [6,10,13-16]. Interestingly, eggs with JC exposed to the 9.4 T field of our magnet are visually indistinguishable from those of the kind depicted in Fig. 1A. However, cells without JC that were subjected to fields above 0.5 T showed a two-phase pigment redistribution with respect to the zero-field controls: In the first phase (TBE I), the white band characteristic of stage VI oocytes and fertilizable eggs concentrically descended from its usually equatorial position towards the vegetal pole (Fig. 1B), while the second phase (TBE II) is characterized by a tongue of this white band moving towards the animal pole (Fig. 1C), with a final arrangement (Fig. 1D) reminiscent of the seam topology of a tennis ball (Fig. 1E). This upward moving tongue was always aligned with the main axis (z) of the magnetic field but randomly oriented in the x-y plane (Fig. 1F). The TBE appeared irreversible and was exclusive to but ubiquitous in dejellied field-exposed fertilizable eggs.

**Figure 2:**
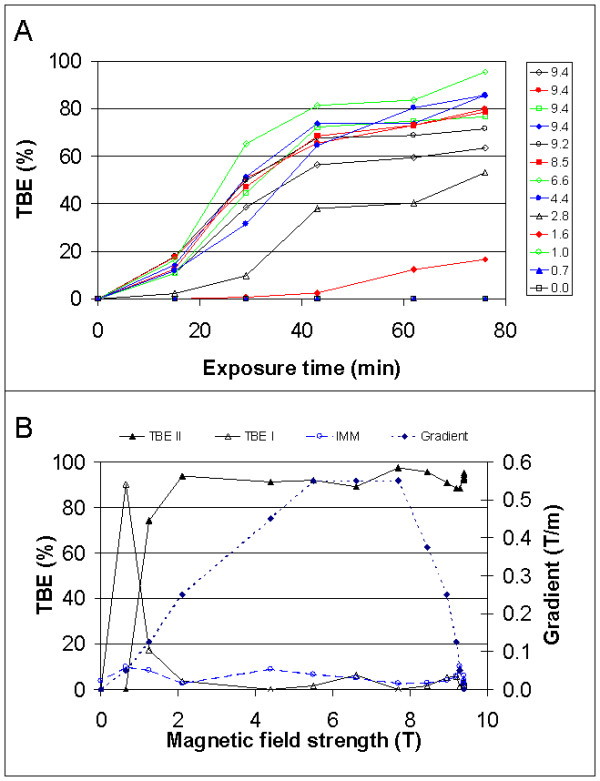
**Magnetic field dependence of the TBE. **The frequency of Tennis Ball Effect (TBE) in two populations of cysteine-dejellied fertilizable *Xenopus *eggs as a function of field strength, gradient strength, and magnet exposure time. (A) Temporal evolution of TBE II at different field strengths (in T). The diagram only gives field exposure times. The total duration of the experiment was about 50 min longer. Each individual point in the diagrams represents a group of about 150 cells. The standard deviation in TBE count, as determined from the four Petri dishes positioned at 9.4 T field strength in the center of the magnet, was 12.3 %. A total of about five percent of the eggs obtained from one ovulation were nekrotic. These were not considered when calculating TBE percentages. (B) TBE percentages after 109 min of continuous magnetic field exposure as a function of field strength and field gradient strength. The TBE counts were all performed after fixation of the eggs, and the standard deviation in the four central dishes was 0.7 % for TBE I and 1.2 % for TBE II. IMM = percentage of immature oocytes present in the dish.

In order to monitor the temporal evolution of the TBE, we took the cells out of the magnet at regular intervals (after 15, 29, 42, 63 and 76 min of cumulative exposure time) and counted the TBE frequency in the individual dishes (cf. Fig. 2A). As the distinction between TBE I, TBE II and no TBE requires closed inspection of each individual cell and is thus time-consuming, only the easily recognizable late TBE II were considered, so that the rack could be repositioned in the magnet after about 10 min. The results reveal that the TBE II frequency depends on exposure time in a sigmoidal way and that a critical threshold just above 1 T is required for TBE II to occur, while all groups exposed to field strengths above 3 T reached essentially the same TBE II percentage after 76 min of exposure to the field. Interestingly, the eggs placed between 4.4 T and 8.5 T – i.e. those exposed to the highest gradients of the static magnetic field – showed a tendency to develop TBE II more quickly and more frequently, though this was not significant due to the relatively high standard deviation of the TBE counts.

To eliminate the possibility that the repetitive insertion and removal of the rack exerts additional gradient-induced stress on the eggs, another experiment was performed in which they were continuously kept in the magnet for 109 min and then directly fixed. To further disambiguate between potential field strength and field gradient strength effects, pairs of Petri dishes with eggs were distributed to storeys of the rack such that both were placed at equal (or, in one case, similar) gradient strengths but one of them at a high, the other at a low field strength. Field strength and gradient strength could not be varied independently in our setup. The results (cf. Fig. 2B) show that although differences exist in TBE percentages between high field and low field at gradient strengths below about 0.2 T/m, these mainly reflect the transition between TBE I and TBE II and do not affect the sum of both TBE percentages. TBE II generally requires just over 1 T to occur, independent of gradient strength, whereas the threshold for TBE I was about 0.5 T. The difference in TBE II percentages between the experiments in Fig. 2A and Fig. 2B can be accounted for by the inclusion of early TBE II in the latter, while the higher standard deviations in Fig. 2A result from the impossibility of detailed inspection of individual cells due to time limits, which was not the case with the fixed samples.

## Discussion

The observed reorganization of the egg cortex supports the initial hypothesis derived from the literature survey (cf. Table 1) – the interplay between the JC and its underlying extra- and intracellular layers (the vitelline envelope and the plasma mambrane, respectively) mediate magnetic field effects in *Xenopus laevis *eggs.

The pigmentation – melanin granula closely linked to the cortex [16] – served us as a visual indicator for this cortical reorganization. However, the melanin might well be responsable for the effect, since it resembles vertebrate pigments discussed in relation to magnetoreception at Earth's field strength [18].

As for *Xenopus*, the involvement of pigments in such rearrangements is also compatible with earlier reports of increased pigmentation anomalies in tadpoles subjected to static fields of 1 T [6]. The occurence of TBE in *all *fertilizable eggs without JC at higher field strengths points at a passive reaction to the magnetic field and suggests the involvement of structures or pathways in the oocyte that are not present before maturation and normally kept in place by the JC.

The cortical rearrangements leading to the TBE probably go along with a redistribution of sperm receptors, which might impede fertilization. However, this could not be tested, as fertilization requires the JC [12], but experiments are under way to clarify whether embryos developing with or without JC show any difference due to magnetic field exposure.

The way in which the JC was removed could also influence the pronounciation of magnetic field effects. Five major approaches have been proposed to achieve it in a way useful for further biological studies of the eggs [13]: Mechanial removal, UV irradiation, alkaline or enzymatic digestion or disulfid-reducing reagents. The first one is too time-consuming for the thousands of eggs necessary for our experiments, and the following three do not specifically act on the JC. This problem concerns the last group as well [6,10,13-16] but we chose cysteine dejellying because it provides a relatively soft approach [13]: It can reliably be stopped before attacking the vitelline envelope. Possibly, though, cysteine actions beyond JC lysis might contribute to the TBE, and further studies should seek to incorporate dejellying mechanisms in the assessment of magnetic field effects.

A detailed understanding of the mechanisms underlying such effects in model systems like *Xenopus *can provide better estimates of possible biological limitations on the applicability of high magnetic fields to cells, tissues and organisms, including humans. In this respect, it is important to note that the minimum field strengths required for TBE I onset and for TBE II saturation, respectively, coincide with the current lower and upper limits of typical clinical magnetic resonance studies [19].

## Conclusion

Fertilizable eggs of *Xenopus laevis *are susceptible to magnetic fields of clinically relevant strengths if and only if deprived of their surrounding jelly layers. These observations suggest that further research on routine or long-term exposure of pigmented biological tissue to strong static magnetic fields is necessary and that *Xenopus *oocytes, eggs and embryos could serve as a suitable test system.

## Authors' contributions

DM conceived of the study, reviewed the literature, participated in the experiments and analyses, and wrote the paper. JWJ designed and performed the experiments, analyzed the data and assisted in drafting the manuscript. HPR took care of the animals, participated in the experiments and analyses, and wrote the paper. All authors read and approved the final version of the paper.
